# Expresión de alfa sinucleína en sangre y su relación con el estreñimiento crónico en población residente en Bogotá, D.C., con problemas de consumo de alcohol

**DOI:** 10.7705/biomedica.4771

**Published:** 2020-06-30

**Authors:** Tania Yadira Martínez-Rodríguez, Mauricio Rey-Buitrago

**Affiliations:** 1 Maestría en Fisiología, Departamento de Ciencias Fisiológicas, Facultad de Medicina, Universidad Nacional de Colombia, Bogotá, D.C., Colombia Universidad Nacional de Colombia Maestría en Fisiología, Departamento de Ciencias Fisiológicas Facultad de Medicina, Universidad Nacional de Colombia BogotáD.C Colombia; 2 Maestria en Genetica Humana, Departamento de Morfologia, Facultad de Medicina, Universidad Nacional de Colombia, Bogota, D.C., Colombia Universidad Nacional de Colombia Maestria en Genetica Humana, Departamento de Morfologia Facultad de Medicina, Universidad Nacional de Colombia BogotaD.C Colombia

**Keywords:** alcoholismo, estreñimiento, alfa-sinucleína, expresión génica, polimorfismo genético, inflamación, Alcoholism, constipation, alpha synuclein, gene expression, genetic polymorphism, inflammation

## Abstract

**Introducción.:**

El consumo excesivo de alcohol resulta en neuroadaptación, neurodegeneración y expresión diferencial de numerosos genes.

**Objetivo.:**

Determinar la relación entre la expresión del gen de la alfa sinucleína (*SNCA*) en sangre, las variantes de nucleótido único (*Single Nucleotide Variant*, SNV) en su región promotora y el estreñimiento crónico en personas con problemas de consumo de alcohol.

**Materiales y métodos.:**

La muestra estuvo conformada por 35 controles y 27 casos, seleccionados según el puntaje obtenido con la herramienta AUDIT. En el diagnóstico del estreñimiento se aplicaron los criterios de Roma IV. La extracción de ácidos nucleicos se hizo a partir de sangre periférica y se evaluó la expresión del gen mediante qPCR, la cuantificación proteica por ELISA y la presencia de SNV en la región promotora del gen por la secuenciación de Sanger.

**Resultados.:**

Se observó sobreexpresión génica relativa de ARNm del gen *SNCA* en el grupo de casos sin relación con el estreñimiento crónico. Se evidenció un riesgo 4,8 veces mayor de presentar estreñimiento en el grupo de casos. Se encontraron nueve variantes de nucleótido simple en un segmento de la región promotora del gen rica en secuencias reguladoras CpG, con frecuencia similar entre los grupos, y se detectó una variante en la posición -2171 que no se encuentra reportada en GenBank para variantes clínicas y cuyo genotipo A/T se relacionó con el incremento de la expresión del ARNm del *SNCA*.

**Conclusión.:**

En personas con problemas de consumo de alcohol se evidenció la sobreexpresión del ARNm de alfa sinucleína*,* lo cual no se relacionó con el diagnóstico de estreñimiento crónico.

## Abstract (en)

**Introduction:**

Excessive alcohol consumption results in neuroadaptation, neurodegeneration, and differential expression of numerous genes.

**Objective:**

To determine the relationship between the expression of the alpha synuclein gene (*SNCA*) in blood, single nucleotide variant (SNV) in its promoter region, and chronic constipation in people with problems of alcohol consumption.

**Materials and methods:**

The sample consisted of 35 controls and 27 cases selected according to the score obtained with the AUDIT tool. For the diagnosis of constipation, the Rome IV criteria were applied. Nucleic acid extraction was performed from peripheral blood and the expression of the gene was evaluated by qPCR, protein quantification by ELISA, and the presence of SNV in the promoter region of the gene by Sanger sequencing.

**Results:**

We observed a relative gene overexpression of *SNCA* mRNA in the case group, which was not related to the diagnosis of chronic constipation. There was 4.8 times greater risk of presenting constipation in the group of cases. Besides, nine single nucleotide variants were found in a segment of the promoter region of the gene rich in CpG regulatory sequences with similar frequency between the groups while a variant was identified in position -2171, which is not reported in GenBank for variants and whose genotype A/T was associated with increased expression of SNCA mRNA.

**Conclusion:**

We evidenced an overexpression of alpha synuclein mRNA in people with problems of alcohol consumption that was not related to the diagnosis of chronic constipation.

A nivel mundial se estima que anualmente el abuso del alcohol causa más de dos millones de muertes y diferentes enfermedades agudas y crónicas, incrementando los costos de la atención en salud y comprometiendo el desarrollo del individuo, la familia y la comunidad ^1^. En Colombia cerca de dos millones y medio de personas consumen alcohol en niveles perjudiciales, lo que representa el 31 % de los consumidores de alcohol y el 11 % de la población entre los 12 y los 65 años ^2^.

El alto consumo de alcohol causa neurotoxicidad y neuroadaptación ^3^^-^^7^^)^ .Se ha postulado que la proteína alfa sinucleína (SNCA*)* está implicada en la sinapsis, la plasticidad neuronal y diversas funciones en las neuronas dopaminérgicas, lo cual la vincula con el sistema de recompensa cerebral que se ve alterado en las adicciones, especialmente el alcoholismo ^3^. Además, se ha demostrado que el flujo de SNCA adopta la dirección cerebro-sangre como mecanismo regulador ^8^, lo que permite su detección en los fluidos humanos ^9^^-^^16^. En este sentido, los estudios en este campo reportan la expresión diferencial del gen que codifica para dicha proteína asociada con la búsqueda y el deseo compulsivo de alcohol (*craving*) ^17^; asimismo, en los estudios en ratas y primates no humanos se ha evidenciado que la sobreexpresión de alfa sinucleína en sangre resulta del consumo prolongado de alcohol ^18^^,^^19^, lo que ha permitido concluir que la elevación de los niveles de ARNm en sangre es común en humanos, roedores y primates y que la alfa sinucleína podría ser útil como biomarcador periférico de alcoholismo crónico ^17^^-^^19^.

Por otro lado, el gen *SNCA* es muy polimorfo, incluida la región promotora participante en el proceso de inicio y regulación de la transcripción; algunas de estas variantes se han relacionado con el alto consumo de alcohol. El polimorfismo Rep1 en la SNCA se asocia con fenotipos de uso, abuso, dependencia y búsqueda de alcohol ^20^; también se han identificado cinco SNV en esta región promotora del gen: rs7678651(C>A), rs7687945 (C>A,T), rs2736995 (A>C), rs2619364 (A>C,G), y rs2301134 (A>G), asociados con el fenotipo del deseo compulsivo (*craving*) de alcohol ^21^^).^

Considerando la influencia de la SNCA en la actividad de la dopamina y las funciones de este neurotransmisor en la motilidad, se han establecido hipótesis sobre una posible relación entre el intestino y el cerebro que postulan que la acumulación de alfa sinucleína se inicia en el intestino y se propaga a través del sistema nervioso entérico hasta el sistema nervioso central ^22^^,^^23^, observándose la disminución de la motilidad intestinal y la aparición de estreñimiento en los modelos animales ^24^^-^^30^.

En este sentido, asumiendo una respuesta similar en humanos y dado el común denominador de la SNCA con el alcoholismo y el estreñimiento, el objetivo del estudio fue determinar la relación entre la expresión de la alfa sinucleína en sangre, las variantes de nucleótido único (SNV) en la región promotora del gen y el estreñimiento crónico en una muestra de población residente en Bogotá con problemas de consumo de alcohol.

## Materiales y métodos

### Muestra de estudio

Se llevó a cabo un estudio exploratorio observacional del tipo de casos y controles con una muestra seleccionada a conveniencia. Participaron inicialmente 306 sujetos residentes en Bogotá convocados por las redes sociales de la Universidad Nacional de Colombia, que respondieron la encuesta inicial para la aplicación de los criterios de inclusión y exclusión, así como el cuestionario *Alcohol Use Disorders Identification Test* (AUDIT) después de firmar el debido consentimiento informado.

Según el puntaje del AUDIT, 115 sujetos cumplían con el correspondiente al grupo de control (≤2 puntos) o al grupo de casos (≥16 puntos), es decir, quienes consumían alcohol en niveles perjudiciales o de dependencia. Estos fueron citados para la segunda fase de toma de las muestras de sangre, a la que asistieron solamente 62 individuos (35 sujetos en el grupo control y 27 en el grupo de casos), que conformaron la muestra final. Se excluyeron aquellos sujetos que consumían fármacos asociados con el estreñimiento, tenían antecedentes de enfermedad hepática o un esquema de tratamiento del alcoholismo o enfermedades psiquiátricas.

### Diagnóstico de estreñimiento crónico

Se aplicaron los criterios de Roma IV para el diagnóstico del estreñimiento ^31^; con el fin de descartar el síndrome de intestino irritable se aplicaron también los criterios de Roma IV para esta enfermedad, naturalmente teniendo en cuenta la información de la historia clínica de los participantes.

### Toma de muestras

Los participantes se abstuvieron de consumir alcohol durante 48 horas como mínimo antes de la extracción de sangre periférica por venopunción. La sangre se recolectó en tubos de tapa lila con EDTA y de los 15 ml obtenidos, seis se destinaron para el aislamiento de células mononucleares y nueve para la extracción del ADN. El plasma obtenido mediante el gradiente de densidad se conservó a -80 °C para su empleo en la prueba de ELISA para proteínas.

### Análisis de la expresión génica de ARNm

Para analizar la expresión de ARNm se aislaron las células mononucleares mediante el gradiente de densidad con el reactivo Histopaque-1077^™^ (Sigma-Aldrich), en tanto que la extracción de ARN total se hizo con el estuche de extracción PureLink RNA mini kit^™^ (Thermo Fischer Scientific) o con el método trizol-cloroformo usando el reactivo RiboZol^™^. Para la reacción de transcripción inversa se utilizó el estuche High Capacity cDNA Reverse Transcription Kit^™^ (Thermo Fischer Scientific) con las siguientes condiciones: 1) a 25 °C durante 10 minutos; 2) a 37 °C durante 120 minutos; 3) a 85 °C durante 5 minutos, y 4) a 4 °C ∞.

Por último, la expresión de los genes se evaluó mediante qPCR utilizando una concentración de 1 ng/µl para todas las muestras de ADNc dentro del rango dinámico estandarizado, con una eficiencia de amplificación del *SNCA* de 2,18, y los iniciadores de diseño propio F: GCCAAGGAGGGAGTTGTGGCTGC y R: TGTTGCCACACCATGCACCACTCC, en una concentración de 0,125 µM, mediante la herramienta *primer-blast* disponible en http://www.ncbi.nlm.nih.gov/tools/primer-blast.

Para el caso del gen normalizador gliceraldehído-3-fosfato deshidrogenasa (*GADPH*), se obtuvo una eficiencia de 1,98 y se utilizaron los iniciadores estándar F: CACCAGGGCTGCTTTTAACTCTGGTA y R: CCTTGACGGTGCCATGGAATTTGC en una concentración de 0,25 µM. Además, se utilizó una muestra calibradora interplaca obtenida de una mezcla de iguales proporciones de ADNc del grupo de casos y del de controles. Para la reacción se usó el estuche Kit Luna Universal qPCR Master Mix^™^; cada muestra se procesó por triplicado (técnico) bajo las siguientes condiciones: 1) un ciclo de desnaturalización inicial a 95 °C durante 300 segundos; 2) 45 ciclos de desnaturalización a 95 °C durante 15 segundos y una amplificación a 65°C durante 30 segundos; 3) un ciclo de fusión a 95 °C durante 10 segundos, a 65 °C durante 60 segundos y a 97 °C durante 1 segundo; 4) un ciclo de enfriamiento a 37 °C. Por último, con los niveles de eficiencia de cada gen y los valores de Cq obtenidos se hizo la conversión a la tasa de expresión.

### Ensayo de inmunoabsorción ligado a enzimas (ELISA)

Para la cuantificación de proteínas se utilizó el estuche Alpha-synuclein (SNCA) (Human) ELISA^™^ de BioVision Incorporated, con una sensibilidad de <9,375 pg/ml y un rango de detección entre 15,6-1.000 pg/ml. La lectura se hizo a 450 nm con una ventana de tiempo de 20 minutos en un equipo Multiskan FC^™^ de Thermo Scientific. Cada muestra se procesó por triplicado y la curva estándar se preparó con una serie de 8 diluciones de 300 µl y concentraciones de 1.000, 500, 250, 125, 62,5, 32,1 y 15,6 pg/ml.

### Evaluación de variantes de nucleótido único

La extracción de ADN se hizo mediante la técnica de precipitación salina (*salting out*). Para la identificación de las SNV de la región promotora del *SNCA* se empleó PCR convencional con los iniciadores de diseño propio empleando la herramienta *primer-blast* disponible en http://www.ncbi.nlm.nih.gov/tools/primer-blast. La secuencia de los iniciadores fue la siguiente:

F: CCGCTTGTTTTAGACGGCTG y R: GTCACGAGCACTCTTGTGGA, para un segmento de 561 pb comprendido entre los nucleótidos -1738 y -2299 de la región promotora del gen.

Después de la PCR, se purificaron los productos con acetato de sodio 3M y etanol absoluto para luego hacer la secuenciación con el método de Sanger empleando el iniciador directo, por lo que se obtuvo la secuencia de la hebra no codificante. Los electroferogramas se visualizaron con el programa BioEdit Sequence Alignment Editor disponible en http://www.mbio.ncsu.edu/BioEdit/bioedit.html, y el alineamiento de las secuencias obtenidas con la secuencia de referencia se hizo con la herramienta BLAST2 disponible en https://blast.ncbi.nlm.nih.gov/Blast.cgi.

### Análisis de datos

El análisis estadístico se hizo con los programas SPSS^™^ (SPSS Inc., Released 2009, PASW Statistics for Windows, version 18.0; Chicago: SPSS Inc.) y GraphPad Prism^™^ (GraphPad Software, Inc). Para determinar la distribución de datos se utilizaron las pruebas de Shapiro Wilk y de Kolmogorov-Smirnov. El nivel de significación se estableció en α=0,05 y todas las pruebas se realizaron a dos colas. Se emplearon, asimismo, las pruebas de Mann-Whitney, ANOVA de dos factores con test *post hoc* de Bonferroni, de Friedman y el test exacto de Fisher y el de ji al cuadrado.

### Consideraciones éticas

El presente estudio fue aprobado por el Comité de Ética de la Facultad de Medicina de la Universidad Nacional de Colombia mediante acta de evaluación 014-224B-17 del 28 de septiembre de 2017. Todos los sujetos aceptaron participar voluntariamente en el estudio y firmaron un consentimiento informado.

## Resultados

### Descripción de los sujetos de estudio

La media de la edad de los participantes fue de 24 ± 4 años para el grupo de control y de 23 ± 4 años para el grupo de los casos. El porcentaje de mujeres fue de 46,8 %. El universo incluyó a personal vinculado a la Universidad Nacional de Colombia y mayoritariamente a estudiantes universitarios.

Tomando como referencia las unidades de bebida estándar y el volumen de un trago por bebida, en un día habitual de consumo de alcohol los hombres del grupo de casos consumían en promedio 192 g y las mujeres 244 g, en tanto que los hombres del grupo de control consumían en promedio 23 g y las mujeres, 37 g.

### Expresión del ARNm del SNCA en células mononucleares de sangre

**Figura 1:**
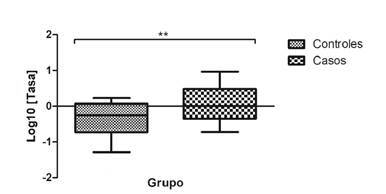
Grado de expresión de ARNm del gen *SNCA* en células mononucleares de sangre periférica. Los valores se presentan en el diagrama de caja con un rango de percentil de 10 a 90; la línea representa la mediana.

Debido a algunas limitaciones técnicas, en este ensayo se analizó una muestra de 25 sujetos del grupo control y 25 del grupo de casos. La tasa de expresión se obtuvo mediante los valores de Cq y se evidenció un incremento en la expresión del ARNm del *SNCA* en el grupo de casos estadísticamente significativo comparado con el grupo de control (p=0,0099) (figura 1).

La expresión génica por sexos presentó una media de 0,465 en las mujeres del grupo de control y de 1.208 en las del grupo de los casos; en los hombres del grupo de control fue de 0,378 y de 1.346 en los del grupo de los casos.

### Cuantificación de la proteína SNCA en plasma

**Figura 2:**
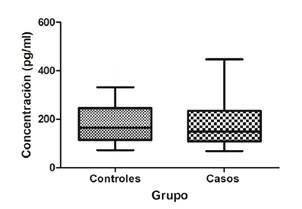
Concentración de la proteína SNCA en plasma sanguíneo. Los valores se presentan en el diagrama de caja con un rango del percentil de 10 a 90; la línea representa la mediana.

Se empleó el plasma de 34 sujetos del grupo control y de 27 del grupo de casos. El valor promedio en los niveles de la proteína en plasma fue mayor en el grupo de casos (199,3 pg/ml) que en el de controles (180,9 pg/ml), sin diferencias estadísticamente significativas entre los dos grupos (p=0,7112) (figura 2).

p =0,7112 en la prueba de Mann-Whitney; n=34 sujetos en el grupo de control y n=27 en el grupo de casos

### Frecuencia de SNV en la región promotora del gen SNCA

Se evaluaron 31 sujetos del grupo de control y 25 del grupo de casos. En general, no se observaron diferencias estadísticamente significativas entre los grupos en ninguna de las nueve variantes estudiadas en el segmento de la región promotora del *SNCA*.

 La secuenciacion de ADN se determino en la hebra no codificante y, por lo tanto, complementaria de los reportados en las bases de datos. De las variantes previamente reportadas y relacionadas con el consumo de alcohol, se encontro una mayor frecuencia en el genotipo [C/C] para la variante rs2619363 (G>C, T), seguido del genotipo heterocigoto [C/A], sin registrarse diferencias estadisticamente significativas (p=0,77). Para la variante rs2301134 (A>G) las frecuencias se distribuyeron en los tres genotipos [C/C], [C/T] y [T/T] con mayor frecuencia el genotipo heterocigoto [C/T] en los controles, aunque sin diferencias estadisticamente significativas (p=0,11) (cuadro 1).

**Cuadro 1:** Frecuencia de genotipos y alelos de diferentes SNV de la región promotora del gen *SNCA* (-1738 y -2299) secuenciados en la hebra no codificante

SNV	n	Genotipo % (n)			Alelos % (n)	
rs2619363 (-2229)		C/C	C/A	A/A	C	A
Controles Casos p<0,05	27 24	63 (17) 62,5 (15)	33,3 (9) 29,2 (7) 0,77	3,7 (1) 8,3 (2)	80 (44) 20(11) 77,1 (37) 22,9(11) 0,72	
rs542037441 (-2195)		C/C	C/A	A/A	C	A
Controles Casos p<0,05	31 25	100 (31) 100 (25)	0 0 >0,99	0 0	100 (62) 0 100 (50) 0 >0,99	

rs989496677 (-2185)		G/G	G/A	A/A	G	A	
Controles Casos p<0,05	31 25	100 (31) 100 (25)	0 0 >0,99	0 0	100 (62) 0 100 (50) 0 >0,99		

SNV (-2171)		T/T	T/A	A/A	T	A	
Controles Casos p<0,05	31 25	16,1 ^5^ 4,4 (1)	83,9 (26) 96,0 (24) 0,14	0 0	58,1 (36) 41,9 (26) 52 (26) 48 (24) 0,52		

rs927159023 (-2159)		C/C	C/G	G/G	C	G	
Controles Casos p<0,05	31 25	100 (31) 100 (25)	0 0 >0,99	0 0	100 (62) 0 100 (50) 0 >0,99		

rs924048519 (-2141)		C/C	C/G	G/G	C	G	
Controles Casos p<0,05	31 25	100 (31) 100 (25)	0 0 >0,99	0 0	100 (62) 0 100 (50) 0 >0,99		

rs2301134 (-2127)		C/C	C/T	T/T	C	T	
Controles Casos p<0,05	31 25	19,4 ^6^ 32 (8)	67,7 (21) 40 (10) 0,11	12,9 (4) 28^7^	53,2 (33) 46,8 (29) 52 (26) 28 (24) 0,90		

rs950036657 (-2120)		C/C	C/T	T/T	C	T	
Controles Casos p<0,05	31 25	100 (31) 100 (25)	0 0 >0,99	0 0	100 (62) 0 100 (50) 0 >0,99		

rs916862395 (-2111)		C/C	C/G	G/G	C	G	
Controles Casos p<0,05	31 25	100 (31) 100 (25)	0 0 >0,99	0 0	100 (62) 0 100 (50) 0 >0,99		

 Asimismo, las seis variantes previamente identificadas en el segmento analizado, rs542037441 (G>T), rs989496677(C>T), rs927159023 (G>C), rs924048579 (G>C), rs950036657 (G>A), y rs916862395 (G>C,T), resultaron ser monomorfas del tipo homocigoto, [G/G], [C/C], [C/C], [C/C], [G/G] y [C/C], respectivamente, en los dos grupos evaluados y en el 100 % de los participantes en el estudio.

 Por otro lado, en el estudio se detectó con gran frecuencia en los dos grupos una SNV en la posición -2171 de la región promotora del gen *SNCA*, la cual no se encuentra reportada en GenBank (NCBI). Dicha variante se observó con mayor frecuencia en el genotipo heterocigoto [T/A] en los dos grupos, sin diferencia estadísticamente significativa entre ellos (p=0,14). 

### Presencia de estreñimiento crónico en la población seleccionada

**Figura 3.**
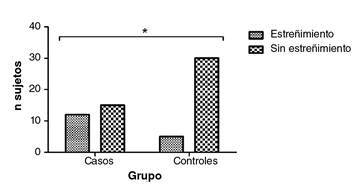
Presencia de estreñimiento crónico en los sujetos de investigación. Los valores corresponden a la frecuencia *p=0,011en la prueba exacta de Fisher; OR=4,8, (IC95% 1,43-16,2); grupo de controles: n=5 con estreñimiento y n=30 sin estreñimiento. Grupo de casos: n=12 con estreñimiento y n=15 sin estreñimiento

Con base en el algoritmo para el diagnóstico del estreñimiento crónico, se encontraron diferencias estadísticamente significativas en cuanto a la presencia de estreñimiento crónico por grupos de consumo de alcohol (p=0,011), observándose un riesgo 4,8 veces mayor de presentar estreñimiento en las personas con problemas de consumo de alcohol (OR=4,8; IC: 1,43-16,2) (figura 3). Se aplicaron los criterios diagnósticos de Roma IV para el síndrome de intestino irritable con el fin de descartar alguna relación entre la presencia de síntomas típicos del estreñimiento crónico y los del síndrome.

### Asociación entre el nivel de proteína SNCA y la prevalencia de estreñimiento crónico

Se evaluaron 34 sujetos en el grupo de control y 27 en el grupo de casos. En general, no se observó una relación entre el diagnóstico de estreñimiento crónico y el consumo de alcohol como factores separados, ni en su interacción sobre el nivel de proteína SNCA (p=0,982).

### Asociación entre la expresión del gen SNCA en forma de ARNm y la prevalencia de estreñimiento crónico

**Figura 4:**
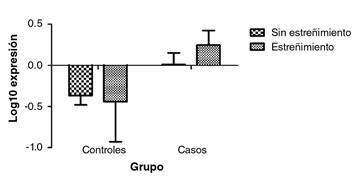
Expresión del gen *SNCA* según el diagnóstico de estreñimiento crónico. Los valores corresponden a la media. p=0,460, en la prueba de ANOVA de dos factores. Grupo de controles: n=3 con estreñimiento y n=22 sin estreñimiento. Grupo de casos: n=11 con estreñimiento y n=14 sin estreñimiento

Se analizaron 25 sujetos en cada grupo de estudio. Se observó un incremento de la expresión del *SNCA* en los sujetos con elevado consumo de alcohol y diagnóstico de estreñimiento crónico (figura 4); no obstante, no se encontró una diferencia estadística significativa para esta interacción (cuadro 2).

**Cuadro 2:** Expresión (log10) del gen *SNCA* según el diagnóstico de estreñimiento crónico

	Controles	Casos	Diferencia	Intervalo de confianza	p
Con estrenimiento	-0,4396	0,2463	0,6859	-0,1663-1,538	>0,05
Sin estrenimiento	-0,3667	0,01003	0,3767	-0,07064-0,8241	>0,05

### Relación entre la expresión del gen SNCA y los SNV

**Figura 5:**
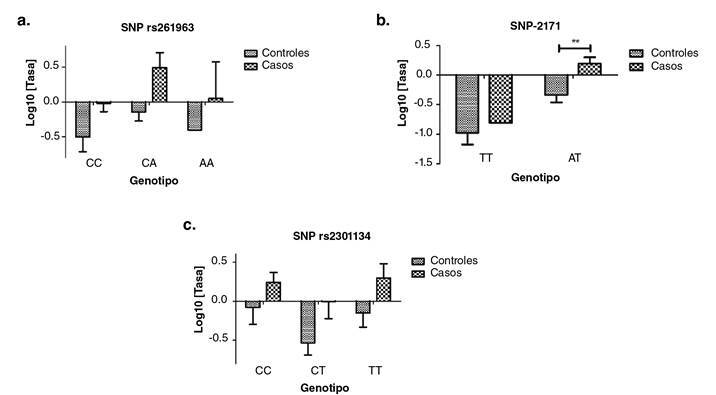
Tasa de expresión del *SNCA* según el genotipo de tres SNV. Los valores corresponden a la media con su respectiva desviación estándar. a. Tasa de expresión según el genotipo del SNV rs261963, p=0,920. b. Tasa de expresión según el genotipo del SNV -2171, p=0,581 en la prueba post hoc y ** p<0,01 para el genotipo AT en los dos grupos. c. Tasa de expresión según el genotipo del SNV rs2301134, p=0,875. n=34 sujetos en el grupo de control y n=27 en el grupo de casos

Se analizaron tres variantes del total de nueve, ya que seis fueron monomorfas en la población estudiada. Según la prueba *post hoc* de Bonferroni, la SNV en la posición -2171 registró una diferencia estadísticamente significativa en la expresión del SNCA entre los grupos de casos y controles en los sujetos con el genotipo AT (p<0,01) (figura 5)

## Discusión

En el estudio se utilizó la herramienta AUDIT validada internacionalmente por la Organización Mundial de la Salud (OMS) ^32^ y, en Colombia, por Campo, *et al.*^33^, y por Ospina, *et al.*^34^. Según la OMS, los criterios de AUDIT establecen que el consumo perjudicial corresponde a 40 g diarios en mujeres y más de 60 g diarios en hombres, en tanto que el consumo de dependencia es de más de 60 g en cualquiera de los sexos ^35^. Al comparar estos criterios con lo reportado en un día habitual de consumo por los participantes en el estudio, se observó en el grupo de casos un consumo muy superior al establecido.

La expresión de ARNm del gen *SNCA* en células mononucleares de sangre periférica fue significativamente mayor en el grupo de casos (p=0,0099), lo que coincide con lo reportado por Walker, *et al.*^19^, en su evaluación de la expresión de ARNm del gen *SNCA* mediante microarreglos en primates alcohólicos que consumieron alcohol libremente durante 18 meses, lo que resultó en un incremento 3,21 veces mayor de los niveles de ARNm en sangre periférica en comparación con los controles (p<0,0001) ^19^.

Es importante tener en cuenta que, en el presente estudio, los sujetos del grupo de casos se abstuvieron de consumir durante las 48 horas previas a la extracción de sangre, a diferencia de los primates no humanos del estudio mencionado, que dispusieron de alcohol hasta el último momento para medir así el efecto crónico y el agudo. Por ello, es posible que, en este caso en particular y bajo las mismas condiciones, el nivel de expresión en humanos se hubiera incrementado.

En otros estudios con humanos, se ha evaluado la expresión génica del *SNCA* en sangre con un periodo de abstinencia de 24 a 72 horas y se ha registrado un aumento significativo de la expresión en el grupo de las personas con alcoholismo (p=0,021) (17) y, aunque el estudio de Bönsch, *et al.*^36^, se hizo únicamente en hombres y en el presente en el grupo de casos había 17 hombres y ocho mujeres, se observó un aumento de la expresión de 1,1 veces en los hombres comparados con las mujeres.

Por otro lado, aunque la concentración de la proteína en plasma aumentó en el grupo de casos (199,3 pg/ml) con respecto al de control (180,9 pg/ml), las diferencias no fueron estadísticamente significativas (p=0,07112), lo que contrasta con lo evidenciado por Bönsch, *et al.*, quienes reportaron un aumento significativo de la proteína en personas con abstinencia de 24 a 72 horas (14,33 ng/ml), comparadas con los controles sanos (5,92 ng/ml) (p<0,0001) ^36^.

En el presente estudio, el incremento en la concentración de la proteína no fue estadísticamente significativo comparado con los niveles de expresión del ARNm del *SNCA* por varias razones: en primer lugar, las determinaciones se realizaron en compartimentos diferentes, el ARNm en lisados de células mononucleares de sangre periférica y la proteína en plasma, por lo que no necesariamente tendrían que ser concordantes, como se ha demostrado en diferentes tejidos y regiones cerebrales ^4^^,^^8^^,^^9^. En segundo lugar, las diferencias podrían deberse a mecanismos de regulación postranscripcionales que impiden la traducción eficaz del ARNm ^37^^,^^38^; tan es así que los cambios postranscripcionales del ARNm del *SNCA* están regulados por la presencia de varios microARN, especialmente el mir-7 y el mir-153, que se unen a la región 3^’^-UTR del *SNCA* y cuya expresión es inversamente proporcional a la expresión del ARNm y la proteína ^39^^,^^40^.

En cuanto a las nueve variantes identificadas de nucleótido único, los hallazgos previos han informado de una asociación entre la presencia de la variante rs2619363 y el fenotipo de deseo compulsivo de alcohol (*craving*) (p=0,01); sin embargo, en el presente estudio no se observó dicha diferencia (p=0,77). En cuanto a la variante rs2301134, aquí no se encontraron diferencias entre las personas en abstinencia durante 48 horas como mínimo y los sujetos de control (p=0,11), al igual que se registró en el estudio de Foroud, *et al*., quienes no encontraron relación entre este SNV y el fenotipo de deseo compulsivo de alcohol (p=0,69) tan estrechamente vinculado con la dependencia ^21^, el cual se da en periodos de abstinencia. Las variantes monomorfas identificadas en la presente muestra tuvieron una frecuencia similar a la reportada mundialmente, sin evidencia de una posible vinculación con el consumo de alcohol.

Por otro lado, en el presente estudio se identificó una SNV en la posición-2171 del promotor del gen *SNCA* con una mayor frecuencia del genotipo heterocigoto [A/T] en los dos grupos evaluados. Esta SNV no se encuentra reportada en GenBank (NCBI) como variante, por lo que su análisis puede ser de utilidad para futuras investigaciones. Esta variante de SNV en -2171 presentó una diferencia significativa para el genotipo heterocigoto [A/T], observándose un incremento de la expresión génica de ARNm en el grupo de los casos (p<0,01). No se encontraron estudios previos que evaluaran la relación entre la expresión del gen y las SNV mencionadas en el promotor. Es importante recordar que el promotor es la región del gen donde se ensambla la maquinaria de la transcripción y que los cambios en los nucleótidos pueden aumentar o disminuir la afinidad por los factores proteicos participantes y, por lo tanto, afectar la tasa de transcripción. En este caso, la SNV -2171 [A/T] podría estar relacionada con ese aumento en la expresión de SNCA en el grupo de casos.

Por otro lado, la presencia de estreñimiento se asoció con un riesgo 4,8 veces mayor en sujetos con problemas de consumo de alcohol (p=0,011); sin embargo, no se evidenció relación con la sobreexpresión de SNCA. Debe tenerse en cuenta que los estudios previos se hicieron en un modelo animal con modificación genética para sobreexpresar ARNm e incrementar los niveles proteicos de SNCA ^24^^-^^30^. En dichos modelos animales, se ha observado una reducción en el número de bolas fecales, un aumento de 2,2 veces en el tiempo de expulsión, un contenido fecal 2,9 veces mayor y una alteración de los estímulos para la defecación correspondiente al estreñimiento ^27^^,^^28^^,^^41^^,^^42^, que posiblemente se relacionen con lo postulado por Sharma, *et al*., en el sentido de que la presencia del estreñimiento se explicaría por la disbiosis que afecta el epitelio del colon, lo que permite que los agregados de SNCA puedan infiltrarse a través del sistema nervioso entérico hasta llegar al sistema nervioso central ^43^.

En el presente estudio observacional, se evidenció una diferencia en la expresión del ARNm del *SNCA* mas no en la proteína, que por su acumulación y agregación causa los efectos motores y no motores (entre ellos el estreñimiento) ya descritos para las llamadas alfa sinucleopatías. Por las tasas de transcripción observadas es poco probable que existan agregados de SNCA (se requieren grandes concentraciones) que puedan infiltrarse en el epitelio intestinal y causar síntomas relacionados con el estreñimiento.

Hasta donde se sabe, este es el primer estudio en el que se intenta evidenciar esta relación, por lo que se requeriría más investigación *in vivo* e *in vitro* de tipo observacional y de intervención, con diseños experimentales orientados a determinarla.

El consumo crónico de alcohol tiene manifestaciones psiquiátricas, psicológicas y biológicas similares a otras enfermedades neurodegenerativas. En este contexto, los resultados del presente estudio no concuerdan con lo observado en los pacientes con enfermedad de Parkinson, en la que también se presentan niveles incrementados de la SNCA y el estreñimiento es frecuente, generalmente antes de que se manifiesten los síntomas clínicos propios de la enfermedad, por lo que se ha asociado la frecuencia de evacuaciones intestinales con el riesgo futuro de padecerla; se ha encontrado que aquellas personas con menos de una evacuación por día presentan un riesgo de 2,7 a 4,5 veces mayor ^44^ y que quienes presentan estreñimiento como antecedente tienen un estadio más grave de la enfermedad ^45^. Sin embargo, aunque tanto en la enfermedad de Parkinson como en el alcoholismo, hay incremento de SNCA y daños cerebrales, es posible que la presencia de estreñimiento en la enfermedad de Parkinson se deba a otros factores diferentes a los del alcoholismo aún no determinados y que no se relacionen con cambios en la permeabilidad del intestino y en la biota intestinal por acción del alcohol, como se ha empezado a esclarecer ^46^, y se relacionan más con otras alteraciones en las vías dopaminérgicas que tienen un papel importante en la motilidad intestinal.

En este estudio, se evidenció la sobreexpresión de ARNm del gen *SNCA* en las células mononucleares de los sujetos con problemas de consumo de alcohol, pero no se encontró una relación con la concentración plasmática de la proteína. Además, en el grupo con alto consumo de alcohol, se registró un mayor riesgo de presentar estreñimiento, aunque sin relación con la expresión diferencial de ARNm del *SNCA*. Por otro lado, no se observó una diferencia estadísticamente significativa en la frecuencia de las SNV y se identificó una en la posición -2171, no reportada previamente como variante en GenBank, y cuyo genotipo [A/T] parece contribuir al incremento de la expresión génica de SNCA.
